# Continuity of care in the context of a primary health care reform: a follow-up after the Swedish *Patient Choice Reform*

**DOI:** 10.1080/02813432.2025.2527856

**Published:** 2025-07-08

**Authors:** Hannes Kohnke, Andrzej Zielinski, Anders Beckman, Henrik Ohlsson

**Affiliations:** ^a^ Department of Clinical Sciences, Faculty of Medicine, Lund University, Lund, Sweden; ^b^ Blekinge Centre of Competence, Region Blekinge, Karlskrona, Sweden; ^c^ Department of Clinical Sciences, Center for Primary Health Care Research, Lund University, Lund, Sweden

**Keywords:** Continuity of patient care, primary health care, health care utilization, health policy, patient choice, privatization, Sweden

## Abstract

**Background:**

Continuity of care (CoC) is essential for effective primary health care (PHC), yet Swedish PHC has historically exhibited low levels of continuity. The Swedish *Patient Choice Reform* introduced privatization and market-oriented principles into PHC, leading to increased utilization and growing inequities in service use driven by socioeconomic disparities and misalignment with health care needs. However, little is known about its impact on continuity. The aim of this study is to explore long-term effects of longitudinal CoC in PHC within the context of the *Patient Choice Reform*.

**Methods:**

Using register data from Region Skåne (2007–2017), we created three closed cohorts, each capturing three years of PHC utilization. Continuity with GPs was measured using the Continuity of Care Index (CoCI). Quantile regression assessed associations between continuity and individual characteristics, including age, sex, income, education and residence.

**Results:**

Among 322,641 individuals with 7,878,642 general practitioner (GP) visits, median CoCI declined from 0.17 (2007 cohort) to 0.13 (2015 cohort). Higher age, male sex and increased PHC utilization were linked to greater continuity in 2007, but these associations weakened by 2015.

**Conclusions:**

Continuity of care in Swedish PHC declined over time, particularly among older individuals and frequent PHC users. These findings highlight the need to address continuity deterioration in the context of the *Patient Choice Reform*.

## Background

In many countries, including Sweden, primary health care (PHC) can be considered the basis of the health care system, playing a significant role in the improvement of the population’s health [1–3]. An essential aspect of a well-functioning PHC is continuity of care (CoC), which refers to a long-term relationship between physician and patient, regardless of the presence of any specific disease [4]. On the patient level, CoC is associated with factors such as greater patient satisfaction [5], improved health promotion [6], increased adherence to medication [7], reduced hospital use [8,9] and decreased mortality rates [10]. On the organizational level, higher CoC in PHC contributes to greater efficiency within health care organizations and lower costs [11,12]. Despite these benefits, Swedish PHC has been characterized by low CoC for decades [13,14].

The concept of CoC varies depending on the scope and setting of the investigation, and several different definitions have been proposed. Depending on the level of interest, CoC may refer to continuity at the clinic level or at the level of individual health care professionals. In the context of defining and measuring CoC, the term *provider* may refer to either. In the following sections of this article, when used in a policy or management context, *provider* refers to the organizational unit.

An ongoing relationship between patient and health care professional, often referred to as *relational continuity*, is distinctly different from *information continuity* (shared information across different encounters and multiple providers) and *management continuity* (a consistent approach to health care across different providers) [15]. Since measuring relational continuity poses conceptual and practical difficulties, *longitudinal continuity* is often used as a proxy [16]. Longitudinal continuity describes the patient’s opportunity to see the same health care professional over time and can be regarded as a prerequisite for ongoing therapeutic relationships [17].

As a CoC metric, longitudinal continuity is appealing because necessary data are often readily available in practice information systems or health insurance claims data. However, there is a lack of consensus on how to measure longitudinal continuity and at least 17 different claims-based indices for measuring CoC have been described [18]. CoC indices can generally be grouped into three major categories based on how they weigh the following: (1) the density of patient visits, (2) the dispersion of care providers and (3) the sequence of visits to providers [19]. Despite differences in construct, many of these metrics are highly correlated [20,21].

In this article, we examine CoC in the context of Swedish PHC. While funding and provision of health care services in Sweden is organized by 21 independent regions, the state is responsible for the overall health care policy [3]. With no or minimal patient fees, the health care system is mainly tax-funded and offers residents of Sweden universal coverage. Half of all doctor visits are made in PHC and PHC accounts for 20% of total health care expenditure [22]. Typically, PHC centers serve as patients’ entry point into the health care system, and are staffed with nurses, general practitioners (GPs) and to a varying extent, other health care professionals. Although PHC has no formal gatekeeping function in most regions, GPs play an important role in both facilitating access to specialized health care (SHC) and in coordinating care from other parts of the health care system [3]. Compared to other OECD countries, Sweden has a relatively low number of GPs per capita [23] and exhibits lower PHC utilization levels along with access limitations [24].

Swedish PHC has had a significant makeover in recent years where a population-based model has been gradually replaced by a more marketized model [24]. The *Patient Choice Reform* of PHC has been especially influential in this change. Political motivations behind the reform were the objectives of improving PHC accessibility and responsiveness to patient expectations through increased patient choice and competition between PHC providers [24]. The reform consisted of two parts and made it mandatory for regions to offer: (1) patients a choice of PHC provider and (2) free entry to the PHC market for providers that fulfill specified conditions for accreditation. In Region Skåne, the reform standards were implemented on 1 May 2009. Following the reform, the number of PHC centers has increased. The increase, however, has been unevenly distributed since private PHC centers have mainly opened in urban areas or areas where health care needs are lower [25–27]. Furthermore, PHC utilization has increased to a higher degree in individuals with minor symptoms as compared to individuals who are more severely ill [26,27].

Although utilization aspects of the *Patient Choice Reform* have been investigated in both scientific reports and by government and regional agencies, information on how quality measures of PHC utilization have been affected remains limited. Small improvements in subjective quality, i.e. patient satisfaction with care, have been reported [28]. However, no additional benefits in terms of reduced hospitalization rates or emergency visits have been observed in regions where the reform was implemented more ambitiously [29]. On the contrary, areas with a consistently high presence of private PHC providers post-reform exhibited the least favorable trends in avoidable hospitalizations [29].

A key limitation in previous assessments of PHC utilization in the context of the *Patient Choice Reform* is the absence of quality measures directly linked to the examined outcomes [26,27,30–34]. While increased utilization is often assumed to reflect improved access and patient benefit, it does not necessarily equate to better quality of care. The reform’s emphasis on patient choice and provider competition may have contributed to a more fragmented PHC landscape, potentially undermining CoC. As CoC relies on stable, long-term relationships between patients and health care professionals, it may be disrupted in a system that prioritizes rapid access and increased provider options. In this context, a decline in CoC following the reform is a plausible expectation.

Despite the central role of CoC in high-quality PHC, the effects of the reform on this and other quality-related aspects remain understudied, and existing studies have not established a clear direction of impact [28,35,36]. This study therefore aims to investigate long-term changes in individual health care utilization, in terms of longitudinal CoC, over a time frame that overlaps with the implementation of the *Patient Choice Reform*.

## Methods

Using a retrospective study design, administrative data was collected on individuals’ utilization of publicly funded PHC services from Region Skåne – the regional government body responsible for overseeing public health care in Skåne County, Sweden’s third most populous region, with 1.3 million residents. Comprising less than 1% of total health care spending in Sweden [37], data from privately funded health care services were not included.

Beyond patient-related needs, health care utilization is shaped by factors that both predispose individuals to seeking care and enable access to it [38]. In addition to age and sex, which serve as needs and predisposing factors, socioeconomic determinants – such as income, education and civil status – play a significant role in health care utilization. These factors are commonly used by Swedish regions as empirical measures for distributing PHC resources [39]. To account for these influences, individual-level data on demographics (age and sex) and socioeconomic status (SES) (income, education and civil status) were obtained from Statistics Sweden and linked to administrative health care utilization data from Region Skåne. Furthermore, since the degree of urbanicity in an individual’s community impacts service utilization and is particularly relevant in the context of the reform [26,40,41], this variable was also included in the analysis.

### Population

Longitudinal health care utilization data was collected for three consecutive periods: 2007–2009, 2011–2013 and 2015–2017. The first period preceded the introduction of the *Patient Choice Reform*, while the latter two followed it. For each period separately, a cohort was created of all inhabitants with a registered address in Skåne during the given time period. These three cohorts will henceforth be referred to as the 2007 cohort, the 2009 cohort and the 2015 cohort. Individuals were included in a cohort if they met the following two criteria: (1) age between 20 and 75 at baseline and (2) had made a minimum of four GP visits during the three-year period. Age was defined at baseline for each cohort separately (1 January 2007, 2011 or 2015). Individuals under 20 years of age were excluded to allow the use of income and education as a proxy for SES. Individuals over 75 years were excluded from the study due to the increasing prevalence of home care services in this age group, and as data on PHC provision through such services are less reliable. The inclusion criteria regarding the minimum number of GP visits was chosen due to characteristics of the outcome measure, which becomes less meaningful with fewer than four visits [42,43]. For each cohort separately, individuals with a registered address outside of Skåne at some point during the three-year follow-up were excluded from the study.

### Outcome measure

To define longitudinal CoC, we used the Continuity of Care Index (CoCI) first defined by Bice and Boxerman [44]. CoCI is a metric that both accounts for the dispersion and distribution of GP visits a patient has over time and with different GPs [45] and is calculated as: (∑*n_i_* − *N*)/*N*(*N* − 1), where *n_i_* is the number of visits that the patient has with the *i*th GP and *N* is the total visits. CoCI can take on values between zero and one. A value of zero signifies the lowest possible continuity and occurs when a different GP is seen for every visit, and a value of one signifies maximum continuity when the same GP is seen at every visit. In contrast to other frequently used indices, CoCI does not assume patients have a designated GP as a ‘usual provider’ and was, hence, regarded as more suitable to assess CoC in the Swedish PHC setting. Based on all GP visits, CoCI was calculated on the individual level for each three-year period separately.

### Independent variables

In order to assess changes in CoCI over time, a ‘cohort’ dummy variable was created, each of the three ‘cohorts’ representing a consecutive three-year period (2007–2009, 2011–2013 or 2015–2017). PHC utilization was defined as the number of GP visits made during each three-year period separately and categorized into four groups (4, 5–6, 7–8 and >8 GP visits). All other independent variables (age, sex, income, educational level and municipality of residence) were defined at baseline for each cohort separately (1 January 2007, 2011 or 2015).

The income variable was defined at the family level as pre-tax family income, adjusted for family size using consumption units. The total consumption weight for each family was calculated based on a scale developed by Statistics Sweden, where weighting varied according to the age and relationship of each family member to the first adult in the household [46]. Income was categorized into three equal-sized groups within each age category based on the income of the 33rd and 67th percentile. Pre-tax income included earnings from employment, business activities, income transfers (such as unemployment benefits, pension payments or paid sick leave) and capital gains, but excluded returns on capital. Educational level was categorized as elementary school, high school or higher education.

The degree of urbanicity of the municipality of residence was determined based on a classification made by the Swedish Association of Local Authorities and Regions. According to this classification, Swedish municipalities are grouped into nine categories based on structural parameters such as population and commuting patterns [47]. For practical purposes, these nine categories were modified to fit into three groups: (1) large cities (population over 200,000) and medium-sized towns (population over 50,000), (2) small towns (population over 15,000) and commuting municipalities near large cities and (3) rural municipalities and commuting municipalities near medium- and small-sized towns. For simplicity, these groups are referred to as urban, semi-urban and rural.

### Statistical method

Quantile regression models were used to assess the effects of independent variables on CoCI. By quantile regression, the median (or other quantiles) for the outcome variable associated with a set of independent variables can be estimated [48,49]. Quantile regression does not assume normality or homoscedasticity of the underlying distribution of independent variables. Furthermore, quantile regression is considered robust to outliers as it allows for assessing the full distribution of the outcome variable and is hence suitable for assessing outcomes that are not normally distributed or are highly skewed [50].

The effects of the independent variables on CoCI were expressed as beta coefficients, describing the estimated change in outcome variable per unit change in the predictor variable. In the first model (A), only the cohort variable was included. In subsequent models, age group and sex (model B), education, income and municipality of residence (model C), and PHC utilization (model D) were added. To assess how the effects of selected variables (age, sex and number of GP visits) change over time, three interaction variables were complemented to model D separately (model E1, E2, E3).

The statistical analyses in this study were conducted using Stata software V.16.1 (StataCorp LP, College Station, TX). To get robust standard errors in the quantile regression, we used non-parametric bootstrap with the IPM algorithm. We evaluated uncertainty in the estimates using 95% confidence intervals (CIs).

## Results

### Descriptive

In 2007, Skåne County had 820,991 residents aged 20–75 where 322,641 (39%) were included in the 2007 cohort. Corresponding percentages of inclusion for cohorts 2011 and 2015 were 40% and 37%, respectively. Comparably, included individuals were somewhat younger, with higher income and level of education than those individuals who did not meet the inclusion criteria (Supplementary Table 1). In all three cohorts together, a total of 7,878,642 GP visits were recorded.

### Comparing the three cohorts

As displayed in Table 1, the number of included individuals varied slightly between cohorts, while the female-to-male ratio remained consistent across all three cohorts. Small differences in cohort characteristics were recorded in terms of age, education and number of GP visits. The median age rose from 50 years in cohort 2007 to 52 years in cohort 2015, which was due to large birth cohorts in the 1940s. Compared to the 2007 cohort, the total number of GP visits was 8% higher in the 2011 cohort and 5% higher in the 2015 cohort. In all three cohorts, women had a higher number of GP visits compared to men. No notable differences between cohorts were recorded regarding income and municipality of residence.

**Table 1.** Cohort population characteristics.

Cohort		Cohort 2007		Cohort 2011		Cohort 2015	
Sex		Female	Male	Female	Male	Female	Male
Number of individuals (*N*)		234,051	176,717	251,209	188,297	247,375	191,367
Proportion of men (%)			43		43		44
Age (years, median)		51	54	50	55	51	56
Age (% of group)	20–44 years	39.1	33.7	39.2	31.6	38.4	29.6
	45–64 years	40.9	42.9	38.5	41.1	38.0	40.3
	65–75 years	20.0	23.4	22.3	27.3	23.6	30.1
Income (% of group)	Low	31.8	25.4	30.1	24.1	29.7	24.8
	Medium	34.9	33.8	36.3	33.9	36.6	34.0
	High	32.3	40.8	33.4	41.7	33.5	40.9
Education level (% of group)	Primary school	11.8	15.0	9.0	12.1	6.7	9.3
	Secondary school	55.9	59.2	55.7	59.9	55.0	61.6
	Higher education	29.7	24.5	34.3	26.9	37.4	28.0
Municipality of residence (% of group)	Urban	41.0	39.2	42.0	39.4	42.9	40.4
	Semi-urban	52.0	54.5	51.6	53.9	51.1	53.2
	Rural	5.0	5.6	5.2	5.8	4.7	5.2
PHC utilization (% of group)	4 GP visits	20.0	24.9	19.5	25.0	19.9	25.5
	5–6 GP visits	28.5	32.2	28.5	32.2	28.7	32.3
	7–8 GP visits	18.1	17.9	18.4	17.8	18.0	17.4
	>8 GP visits	32.3	25.1	33.6	25.0	33.4	24.9
CoC (median, 25th–75th percentile)		0.17 (0.08–0.30)	0.17 (0.10–0.33)	0.13 (0.06–0.25)	0.17 (0.07–0.30)	0.11 (0.05–0.2)	0.14 (0.07–0.27)

**Figure 1.**
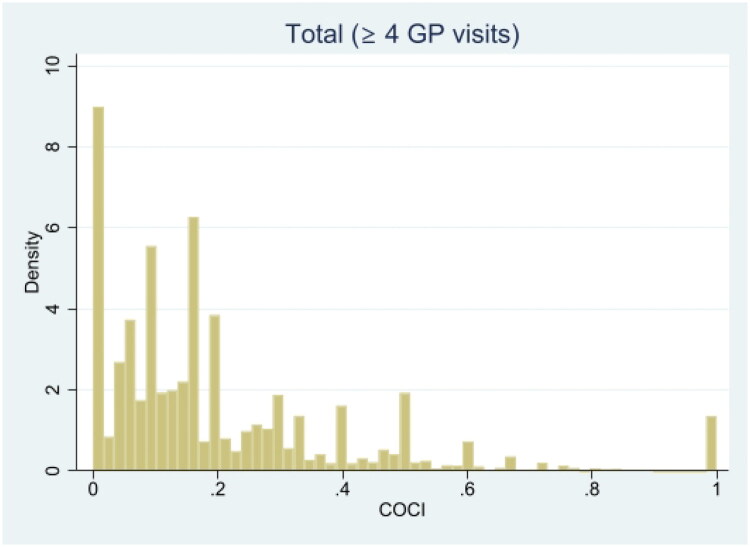
Histogram showing the distribution of Continuity of Care Index (CoCI) across all three cohorts combined. Density on the *Y*-axis represents the relative frequency per unit on the *X*-axis.

The distribution of CoCI was positively skewed in the population (Figure 1) and median CoCI was recorded as 0.17 in the 2007 cohort, 0.14 in the 2011 cohort, and 0.13 in the 2015 cohort.

### Quantile regression

In model A, single effects of the cohort variable on CoCI were analyzed, and compared to the 2007 cohort, CoCI had decreased by −0.024 (95% CI −0.025 to −0.023) and −0.039 (95% CI −0.041 to −0.038) in cohorts 2011 and 2015, respectively (Table 2). These cohort effects remained relatively unchanged when adjusted for age and sex (model B), income, education and municipality of residence (model C), and number of GP visits (model D).

**Table 2.** Quantile regression showing effects of independent variables on Continuity of Care Index (CoCI).

		Model A		Model B		Model C		Model D		Model E1		Model E2		Model E3	
		*β*	*p* Value (95% CI)	*β*	*p* Value (95% CI)	*β*	*p* Value (95% CI)	*β*	*p* Value (95% CI)	*β*	*p* Value (95% CI)	*β*	*p* Value (95% CI)	*β*	*p* Value (95% CI)
Cohort	2007	Ref.		Ref.		Ref.		Ref.		Ref.		Ref.		Ref.	
	2011	−0.024	.000 (–.025, −.023)	−0.030	.000 (–.031, −.029)	−0.030	.000 (–.030, −.029)	−0.033	.000 (–.034, −.032)	−0.023	.000 (–.025, −.021)	−0.025	.000 (–.027, −.024)	−0.004	.000 (–.006, −.002)
	2015	−0.039	.000 (–.041, −.038)	−0.045	.000 (–.046, −.044)	−0.045	.000 (–.046, −.044)	−0.043	.000 (–.044, −.042)	−0.030	.000 (–.031, −.028)	−0.038	.000 (–.040, −.037)	−0.005	.000 (–.007, −.003)
Age group	20–44 years			Ref.		Ref.		Ref.		Ref.		Ref.		Ref.	
	45–64 years			0.045	.000 (.044, .046)	0.045	.000 (.044, .046)	0.036	.000 (.035, .037)	0.046	.000 (.044, .048)	0.037	.000 (.036, .038)	0.038	.000 (.037, .039)
	65–75 years			0.060	.000 (.059, .061)	0.058	.000 (.057, .059)	0.045	.000 (.044, .046)	0.074	.000 (.072, .076)	0.045	.000 (.043, .046)	0.042	.000 (.041, .043)
Sex	Female			Ref.		Ref.		Ref.		Ref.		Ref.		Ref.	
	Male			0.018	.000 (.017, .019)	0.016	.000 (.015, .016)	0.010	.000 (.009, .011)	0.010	.000 (.009, .011)	0.018	.000 (.016, .020)	0.005	.000 (.004, .006)
Education	Primary school					Ref.		Ref.		Ref.		Ref.		Ref.	
	Secondary school					−0.002	.000 (–.003, −.001)	−0.001	.021 (–.003, −.000)	0.000	1.000 (–.001, .001)	−0.001	.114 (–.003, .000)	−0.001	.277 (–.002, .001)
	Higher education					−0.006	.000 (–.007, −.005)	−0.003	.000 (–.004, −.002)	−0.003	.000 (–.004, −.001)	−0.003	.004 (–.004, −.001)	−0.002	.012 (–.003, −.000)
Income	Low income					Ref.		Ref.		Ref.		Ref.		Ref.	
	Middle income					0.001	.000 (.001, .002)	0.001	.001 (.001, .002)	0.001	.074 (–.000, .002)	0.001	.029 (.000, .002)	0.000	.453 (–.001, .001)
	High income					0.004	.000 (.003, .00)	0.003	.000 (.002, .004)	0.004	.000 (.003, .005)	0.003	.000 (.001, .004)	0.001	.004 (.000, .002)
Municipality of residence	Urban					Ref.		Ref.		Ref.		Ref.		Ref.	
	Semi-urban					−0.006	.000 (–.007, −.005)	−0.003	.000 (–.004, −.002)	−0.004	.000 (–.005, −.003)	−0.003	.000 (–.004, −.002)	−0.001	.000 (–.002, −.001)
	Rural					0.004	.000 (.002, .005)	0.003	.000 (.001, .005)	0.003	.003 (.001, .005)	0.004	.001 (.002, .006)	0.002	.013 (.000, .004)
PHC utilization	4 GP visits							Ref.		Ref.		Ref.		Ref.	
	5–6 GP visits							−0.025	.000 (–.026, −.024)	−0.033	.000 (–.034, −.032)	−0.027	.000 (–.028, −.026)	0.006	.000 (.004, .007)
	7–8 GP visits							−0.015	.000 (–.016, −.014)	−0.020	.000 (–.021, −.019)	−0.016	.000 (–.018, −.015)	0.016	.000 (.014, .018)
	>8 GP visits							−0.003	.000 (–.004, −.002)	−0.009	.000 (–.010, −.008)	−0.003	.000 (–.004, −.001)	0.034	.000 (.033, .036)
Interaction between cohort and age group	Cohort 2011 and 45–64 years									−0.013	.000 (–.016, −.011)				
	Cohort 2011 and 65–75 years									−0.035	.000 (–.037, −.032)				
	Cohort 2015 and 45–64 years									−0.016	.000 (–.019, −.014)				
	Cohort 2015 and 65–75 years									−0.041	.000 (–.044, −.038)				
Interaction between cohort and sex	Cohort 2011 and male											−0.012	.000 (–.014, −.010)		
	Cohort 2015 and male											−0.005	.000 (–.007, −.003)		
Interaction between cohort and PHC utilization	Cohort 2011 and 5–6 GP visits													−0.034	.000 (–.037, −.031)
	Cohort 2011 and 7–8 GP visits													−0.036	.000 (–.039, −.033)
	Cohort 2011 and >8 GP visits													−0.038	.000 (–.041, −.036)
	Cohort 2015 and 5–6 GP visits													−0.041	.000 (–.044, −.038)
	Cohort 2015 and 7–8 GP visits													−0.042	.000 (–.045, −.039)
	Cohort 2015 and >8 GP visits													−0.057	.000 (–.059, −.054)

In the full model (D), CoCI increased with age, and compared to the youngest age group, CoCI was respectively 0.036 (95% CI 0.035–0.037) and 0.045 (95% CI 0.044–0.046) higher in age groups 45–64 and 65–75 years. To analyze age effects across cohorts, the full model was complemented with an interaction term between the cohort variable and age (models E1). Across cohorts, the effect of age on CoCI was recorded to decrease. The largest decrease in effect was recorded for the oldest age group where CoCI decreased by −0.041 (95% CI −0.044 to −0.038) in the 2015 cohort as compared to the 2007 cohort.

Sex differences in CoCI were recorded in the full model, and CoCI was 0.010 (95% CI 0.009–0.011) higher in men compared to women. As compared to the 2007 cohort, the sex effect on CoCI decreased slightly in the 2011 cohort and remained unchanged in the 2015 cohort (model E2).

In the full model, the effect of PHC utilization on CoCI was non-linear, and CoCI was lower for groups with 5–6 or 7–8 GP visits as compared to groups with only four, or more than eight, GP visits. This non-linear relationship was explained by large differences in the effect of PHC utilization across cohorts (model E3). In the 2007 cohort, CoCI increased with increasing PHC utilization, and CoCI was 0.034 (95% CI 0.033–0.036) higher in the group with more than eight visits as compared to the group with only four visits. Across cohorts, the effect of PHC utilization on CoCI decreased. The largest decrease was recorded in the group with more than eight GP visits where CoCI decreased by −0.057 (95% CI −0.059 to −0.054) in the 2015 cohort compared to the 2007 cohort.

**Figure 2.**
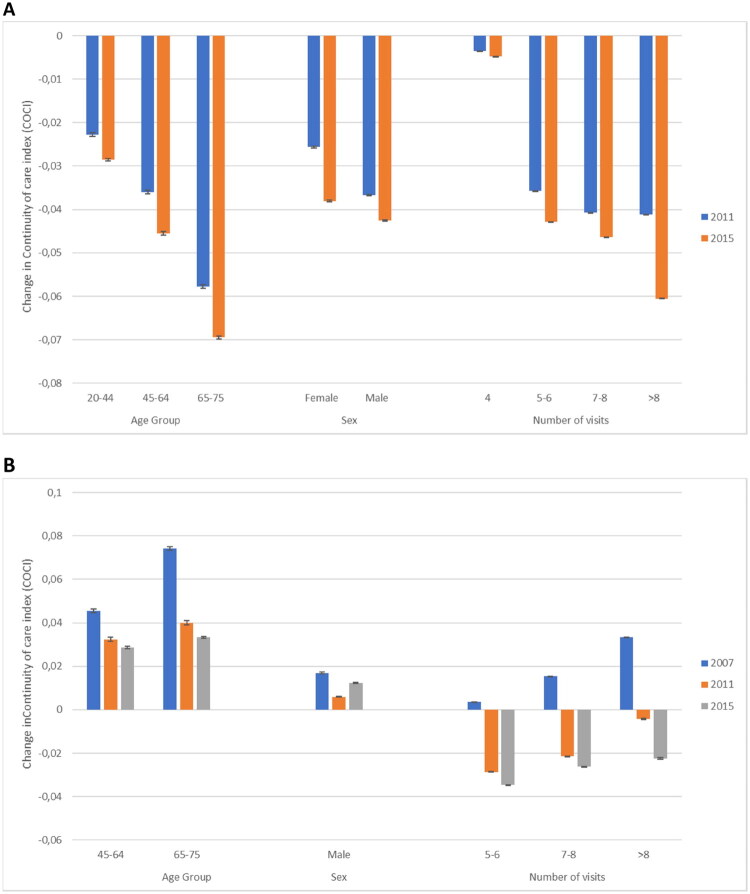
Postestimation predictions of CoCI based on age, sex and PHC utilization. Changes in Continuity of Care Index (CoCI) are based on models E1, E2 and E3. In (A), bars show change in CoCI compared to cohort 2007. In (B), bars show change in COCI compared to the reference group for each variable: age group 20–44 years, female sex, and four annual visits.

Postestimation predictions on change in CoCI based on models E1, E2 and E3 are presented in Figure 2. Here, variable-specific effects of age, sex or PHC utilization were added to cohort effects, in order to get the total change in CoCI across cohorts.

## Discussion

### Principal findings

In this long-term follow-up, examining changes in CoC in PHC in the context of the Swedish *Patient Choice Reform*, we found CoC to decrease over time. Our findings show CoC to be low already prior to the reform in 2007 and to decrease further in the years following the reform. Prior to the reform, CoC was observed to be higher in older individuals or individuals with a higher PHC utilization. As CoC is particularly important for more vulnerable patients [51], this suggests an adequate priority where patients with presumed higher health care needs receive higher CoC. Following the reform, however, the effect of age and PHC utilization on CoC gradually decreased and the subsequent decrease in CoC was comparably higher in older individuals and in individuals with a higher PHC utilization. Sex differences in CoC prior to the reform were small; women had a slightly higher CoCI than men and were reduced further following the reform. Furthermore, SES and municipality of residence had no relevant effects on CoC.

### Interpretation within the context of the wider literature

Published material on CoC in Swedish PHC populations is sparse and comparisons of outcomes between studies should be exercised with care due to the multitude of CoC metrics used and varying definitions regarding follow-up time and minimum number of contacts to inclusion. However, CoCI values recorded in this study are in line with results from a previous study of a PHC population in another Swedish region [9]. When comparing CoC across countries or health care systems, further caution should be exerted due to differences in health care provision and study populations. From an international perspective [12,45,52,53], however, reports of CoC in Swedish PHC stand out as exceptionally low. Explanations for the rather large difference are likely to be many, including a relative shortage of GPs and the tradition of having ‘hospital-centered health care’ [3]. Given the observed decline in CoC, further investigation is needed to explore how CoC can be maintained for populations with greater health care needs despite constraints in human resources. At the system and management levels, approaches to incentivize long-term relationships between patients and health care professionals, to enhance CoC within Swedish patient choice models, also warrant further exploration.

A well-documented effect of the *Patient Choice Reform* is the expansion of PHC provision, primarily in urban areas where most new private PHC centers have been established [25,26,54]. Since CoC partly depends on the availability of GPs, one might expect the increased resource allocation in urban areas to have a positive impact on CoC. However, our results show no differences in CoC across municipalities with varying degrees of urbanization. A possible explanation for this might be that, despite the growing geographical inequity in PHC allocation following the reform, the distribution of GPs between urban and non-urban areas has remained relatively stable over time. Another possibility is that any potential improvement in CoC due to increased GP availability in urban areas is counterbalanced by opposing factors, such as a more fragmented PHC system. Besides the relative shortage of GPs, potential barriers to CoC within Swedish patient choice models require further exploration. Such research should consider how patient choice models may contribute to health care fragmentation in the Swedish PHC system and act as a barrier to achieving CoC. Given the ongoing trend of digitalization [55], incorporating this perspective would be relevant.

### Strengths

Among the strengths of this study are the access to detailed and longitudinal register data on all residents in the region, and the construct of three closed cohorts from which reliable CoC measures could be obtained. Potential imbalances between cohorts could be accounted for in the regression model thus limiting confounding due to differences between cohorts.

### Limitations

Excluding individuals over 75 years of age limits the interpretation of the results, particularly as older adults, due to higher levels of morbidity, are likely to benefit more from CoC than younger age groups. Similarly, excluding individuals with two or three GP visits presents a limitation in assessing CoC among those with lower health care needs. The absence of an objective measure of morbidity limits the ability to differentiate between patients with varying levels of health care needs. Furthermore, we could not explore the role of non-GP health professionals, which may be relevant for patients with higher health care needs. Limiting the interpretation of potential effects of the *Patient Choice Reform* are other factors implicative for PHC utilization that have likely changed during the observation period. Such factors, whether they are on the individual or population level, mandate caution when assuming potential reform effects. A general limitation that applies to all analyses of administrative data is the lack of information about the patient experience. Even though the applied outcome measure in this study is a strong PHC quality measure, the metric shows nothing about the subjective quality of individual GP visits.

### Generalizability

The results of this study may be generalized to other Swedish regions with some considerations. Regional variations in population demographics and health care provision are likely to affect CoC, and a report on CoC by the Swedish Agency for Health and Care Services Analysis showed regional differences in COCI [56]. In this report, Skåne has the third-highest recorded CoCI of all Swedish regions.

## Conclusions

Starting from a baseline of low CoC, this long-term follow-up reveals a further decline in CoC during a period that overlapps with the implementation of the *Patient Choice Reform* in Swedish PHC. Patient groups with a perceived higher need for CoC were recorded with the largest decrease in CoC during follow-up, suggesting an inadequate prioritization of resources. Given the demographic challenge posed by an aging population, further research should focus on how CoC can be maintained for patient groups with greater health care needs, despite limitations in human resources. Considering the potential implications for overall system efficiency, further exploration is needed to understand how patient choice models may contribute to health care fragmentation within the Swedish PHC system and act as a barrier to achieving CoC.

## Supplementary Material

Scandinavian_SupplementaryTable_250522.docx

## Data Availability

The datasets generated and analyzed in this study are available from the corresponding author upon reasonable request.
