# Supplementary material for: Continuity of care in the context of a primary health care reform: a follow-up after the Swedish Patient Choice Reform
Source: Scand J Prim Health Care. 2025 Jul 8;44(1):1–13. doi: 10.1080/02813432.2025.2527856 (PMC12928609; doi:10.1080/02813432.2025.2527856)
Supplement: Scandinavian_SupplementaryTable_250522.docx [file IPRI_A_2527856_SM3415.docx]

**Supplementary Table 1**. Comparison of individuals in study population based on number of GP visits per individual.

| **Cohort** | | | **2007** | | | | **2011** | | | | **2015** | | | |
| --- | --- | --- | --- | --- | --- | --- | --- | --- | --- | --- | --- | --- | --- | --- |
| **Number of GP visits** | | | **1-3 visits** | | **≥4 visits** | | **1-3 visits** | | **≥4 visits** | | **1-3 visits** | | **≥4 visits** | |
| **Number of individuals (N)** | | | 90,127 | | 322,641 | | 94,618 | | 344,888 | | 94,765 | | 333,977 | |
| **Proportion of men (%)** | | | 48.9 | | 41.1 | | 49.4 | | 41.1 | | 48.9 | | 40.4 | |
| **Age (year, median)** | | | 45 | | 50 | | 46 | | **51** | | 48 | | **52** | |
| **Age (% of group)** | **20–44 years** | 48.8 | | 36.9 | | 46.5 | | 36.1 | | 43.1 | | 34.9 | |  |
|  | **45–64 years** | 37.2 | | 41.7 | | 36.8 | | 39.6 | | 36.8 | | 38.9 | |  |
|  | **65–75 years** | 14.1 | | 21.4 | | 16.7 | | 24.4 | | 20.1 | | 26.2 | |  |
| **Income (% of group)** | **Low** | 27.4 | | 29.2 | | 25.5 | | 27.7 | | 24.4 | | 27.7 | |  |
|  | **Medium** | 34.7 | | 34.5 | | 34.2 | | 35.3 | | 34.8 | | 35.5 | |  |
|  | **High** | 37.9 | | 35.8 | | 39.7 | | 36.8 | | 40.3 | | 36.5 | |  |
| **Education level (% of group)** | **Primary school** | 9.3 | | 13.1 | | 7.2 | | 10.3 | | 5.5 | | 7.7 | |  |
|  | **Secondary school** | 55.5 | | 57.3 | | 54.8 | | 57.4 | | 54.6 | | 57.7 | |  |
|  | **Higher education** | 33.7 | | 27.6 | | 36.7 | | 31.2 | | 38.6 | | 33.6 | |  |
| **Municipality of residence (% of group)** | **Urban** | 40.8 | | 40.3 | | 40.8 | | 40.9 | | 40.9 | | 41.9 | |  |
|  | **Semi-urban** | 52.3 | | 53.0 | | 51.8 | | 52.5 | | 51.5 | | 51.9 | |  |
|  | **Rural** | 5.0 | | 5.3 | | 4.9 | | 5.5 | | 5.1 | | 4.9 | |  |
